# Insights into sleep's role for insight: Studies with the number reduction task

**DOI:** 10.2478/v10053-008-0143-8

**Published:** 2013-12-31

**Authors:** Rolf Verleger, Michael Rose, Ullrich Wagner, Juliana Yordanova, Vasil Kolev

**Affiliations:** 1 Department of Neurology, University of Lübeck, Germany; 2 Department of Systems Neuroscience, University of Hamburg, Germany; 3 Charité, University Medicine Berlin, Germany; 4 Institute of Neurobiology, Bulgarian Academy of Sciences, Sofia, Bulgaria

**Keywords:** sleep, insight, implicit learning, explicit knowledge, EEG, ERPs

## Abstract

In recent years, vibrant research has developed on “consolidation” during sleep: To what extent are newly experienced impressions reprocessed or even restructured during sleep? We used the number reduction task (NRT) to study if and how sleep does not only reiterate new experiences but may even lead to new insights. In the NRT, covert regularities may speed responses. This implicit acquisition of regularities may become explicitly conscious at some point, leading to a qualitative change in behavior which reflects this insight. By applying the NRT at two consecutive sessions separated by an interval, we investigated the role of sleep in this interval for attaining insight at the second session. In the first study, a night of sleep was shown to triple the number of participants attaining insight above the base rate of about 20%. In the second study, this hard core of 20% discoverers differed from other participants in their task-related EEG potentials from the very beginning already. In the third study, the additional role of sleep was specified as an effect of the deep-sleep phase of slow-wave sleep on participants who had implicitly acquired the covert regularity before sleep. It was in these participants that a specific increase of EEG during slow-wave sleep in the 10-12 Hz band was obtained. These results support the view that neuronal memory reprocessing during slow-wave sleep restructures task-related representations in the brain, and that such restructuring promotes the gain of explicit knowledge.

## Introduction

In this paper, we will provide a review of our research that has used the number reduction task (NRT) to study insight and its relation to sleep (Lang et al., 2006; Wagner, Gais, Haider, Verleger, & Born, 2004; Yordanova et al., 2008; Yordanova, Kolev, Wagner, Born, & Verleger, 2012; Yordanova, Kolev, Wagner, & Verleger, 2009, 2010).

These studies were conducted within the more general context of research on the role of sleep for learning and memory (Diekelmann & Born, 2010; Stickgold & Walker, 2013). This area of sleep research drew its inspiration from a model of learning and memory by McClelland, McNaughton, and O’Reilly (1995). These authors assumed that memory relies on two stores, intermediate and long-term, and that most of the information acquired during the organism’s period of activity is brought to the intermediate store and then read out to the long-term store during periods of inactivity. One advantage of such two-stage structure would be that new information may be learned without overwriting older information. McClelland et al.’s model identified the intermediate store with the hippocampal system and the long-term store with the neocortex. The period of activity in humans is usually the time of being awake during daytime, and the period of inactivity is during sleep. Indeed, a considerable body of evidence has accumulated in support of this hypothesized active role of sleep for consolidating newly acquired memories (Diekelmann & Born, 2010).

Insight into a hitherto unidentified regularity may be considered a particular type of creative behavior (Dietrich & Kanso, 2010). Insight is characterized by a sudden transition from a state of not knowing to a state of knowing, potentially more appropriate to the problem and in any case having a major impact on the person’s conception of the problem (Hélie & Sun, 2010). According to Hélie and Sun, *insight* may be defined as part of a problem-solving process that proceeds in stages: There is a first, more or less extended encounter with a given task. This first encounter is followed by a period of “incubation”, that is, a period away from deliberative work on the problem, where some beneficial processes may take place. This period may or may not lead to an insight. This insight will then be verified while the task is performed anew. Using these terms, the question whether insight is promoted by sleep may be specified as the question whether insight is promoted when there is sleep during the incubation period following the first encounter with the task.

In order to measure insight in the laboratory, a suitable task is needed that provides for objective, reproducible measures of insightful behavior. Other studies on insight have used explicit, verbal problem solving. For example, participants had to find the word that combines with each of three presented words to form new words (remote associations test; see Cai, Mednick, Harrison, Kanady, & Mednick, 2009; Kounios et al., 2006; e.g., a solution for *pine, crab, sauce* would be *apple*) or had to find solutions to brainteasers (Sheth, Sandkühler, & Bhattacharya, 2009; e.g., there are three on-off light switches, one of the switches controls an incandescent bulb in another room, you can only walk once to check on the light bulb). But the way leading to insight in these tasks is hard to measure because there is no overt behavior while participants are trying to find the solution. An interesting alternative is offered by the serial reaction-time task (SRTT; Willingham, Nissen, & Bullemer, 1989): Participants respond to the appearance of a stimulus at one of several locations by pressing the spatially corresponding key. Unknown to participants, the sequence of locations (and thereby, responses) follows a rule (e.g., by forming a repeating sequence of length 12). Thereby, the question may be studied if and how participants arrive at discovering this regularity, particularly if and how such discovery depends on implicit acquisition of the regularity. In this context, *insight* is defined as getting explicit knowledge of some regularity that might have been implicitly acquired before. Implicit acquisition is indicated by changes in behavior and may be measured on-line during the SRTT, by quantifying the differences of participants’ response times between regular sequences, where some speeding is expected to occur because of the regularity, and interspersed random sequences, where no such speeding is expected. *Explicit knowledge* of the regularity is defined as knowledge that is accessible to consciousness and can be expressed in words. Explicit knowledge may be measured after the task, by asking participants to actively generate sequences of stimuli that may have occurred before.

**Figure 1.**
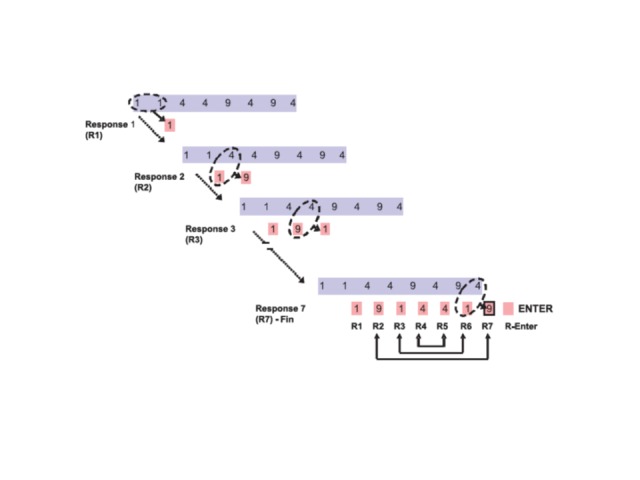
Number reduction task (NRT) illustrated by an example trial. Subjects sequentially transform a given sequence of the three digits (1, 4, and 9) into a new sequence to determine the final result of this trial (Response 7 [R7] – Fin). The hidden task structure implemented in all trials is that the digits used as last three responses mirror the previous three responses (illustrated by the pairwise arrows at the bottom of the figure), which implies that the second response is always equal to the final result (R2 = R7).

We used another task that follows a similar rationale and extends it: the NRT (Rose, Haider, Weiller, & Büchel, 2002; Woltz, Bell, Kyllonen, & Gardner, 1996). In the NRT, unknown to participants, the responses occurring late in each trial are completely predictable from the responses given early in the trial. The task is illustrated in Figure 1 by an example trial. Briefly, in this version as used, for example, by Wagner et al. (2004) and Yordanova et al. (2008), on each trial a string of eight digits is presented, composed of the digits 1, 4, and 9. Strings differ between trials. The final result of a string has to be determined. This may be achieved by sequentially processing pairs of digits from left to right by two simple operations, to be described below, and entering the result of each pair-wise operation on the keyboard. Once entered, the response appears on the screen, forming an expanding response string below the stimulus sequence. The final response is to be highlighted by pressing the “Enter” key. When the “Enter” key is pressed, the response last entered turns blue, and if this final response is correct, the entire display of stimulus and response strings turns blue.

Importantly, unmentioned to participants, all strings were gene-rated according to the same underlying regularity, which, if discerned, allows an early determination of the final result. Specifically, all response sequences have the form A-B-C-D-D-C-B (with each one of the four letters A, B, C, and D representing one of the three digits 1, 4, or 9), that is, the last three responses always mirror the preceding three responses, so that the second response in each trial coincides with the final result. When gaining insight into this regularity, participants can abruptly cut short their sequential responding by pressing the “Enter” key already after the second response (R2 in Figure 1), whereupon the trial is finished and the next trial starts. Note that identifying the predictive power of R2 on R7 does not necessarily imply identifying the fully mirrored structure of R2-R7, R3-R6, R4-R5. On the other hand, being relatively easy to identify, the immediate repetition R4-R5 was supposed to help in identifying the fully mirrored structure, thus to form one way of identifying R2’s predictive power on R7. Further note that, unlike in the SRTT, this regularity is abstract because the actual digit strings and responses change from trial to trial. Thus, the rule cannot simply be discovered on the basis of repeating the same finger movements in each trial. During instruction, participants are informed that the “Enter” key can be pressed whenever the response is the final result. But this instruction is not explicitly emphasized, in order to minimize active problem-solving behavior.

The actual operations used to sequentially process the digit pairs were irrelevant for the major purpose of the task. The requirement to acquire and apply these rules served the welcome side effect of distracting participants form realizing what the task was about. One of two operations had to be applied, depending on whether the digits of a pair were identical or not:

1. The result of two identical digits is the same digit (e.g., 1 and 1 gives 1; R1 in Figure 1).

2. The result of two different digits is the remaining third digit (e.g., 1 and 4 gives 9, as with R2 in Figure 1; 9 and 4 gives 1, as with R3 in Figure 1, etc.).

Thus, like in the SRTT, implicit acquisition of the regularity may be measured by assessing the difference in response times between predictable and unpredictable responses. Moreover, explicit (i.e., consciously accessible) insight into this rule may be measured on-line, rather than only off-line as in the SRTT, because when having discovered the rule, participants can mark their second response already as final result (by pressing the “Enter” key), thereby drastically reducing the number of responses required in the trial and, therefore, having an immediate advantage from their discovery. Having available these two independent measures of implicit acquisition of the regularity, on the one hand, and of explicit insight into this regularity, on the other hand, the following question can now be studied: How these two measures relate to each other and, of particular importance, whether their relation is modified by sleep, specifically whether sleep is needed during the incubation period after some first encounter with the NRT in order to proceed from implicit acquisition to explicit insight.

Our first study to be reported here showed that insight is indeed promoted when there is sleep during the incubation period following the first encounter with the NRT (Wagner et al., 2004). We then investigated separately the causes of the base rate of about 20% of participants who attained insight without the help of sleep, and the causes of the sleep-induced increase of the number of insightful participants. Pursuing the non-sleep branch in a study without an incubation period, task-related event-related electroencephalography (EEG) potentials were measured and turned out to differ from the very beginning between people who would and people who would not later attain insight (Lang et al., 2006). Evidently, the effect of the first encounter with the task is decisively moderated by the mental set taken by the individual participant. This variation may account for the relatively stable percentage of 20% rule-discoverers without sleep. The specific mechanisms additionally working in sleep were investigated in another study (Yordanova et al., 2008; with further analyses reported in Yordanova, Kolev, & Verleger, 2009; Yordanova, Kolev, Wagner, & Verleger, 2009, 2010, 2012; Yordanova, Verleger, Wagner, & Kolev, 2010). The deep-sleep phase of slow-wave sleep (SWS) turned out to be critical in participants who had implicitly acquired the covert regularity before sleep. These participants formed a cluster of their own, and it was in these participants that a specific increase of EEG during SWS in the 10-12 Hz band was obtained. These results support the view that sleep works on implicitly acquired knowledge, and that it is neuronal reprocessing during SWS that lays the foundations for restructuring those task-related representations in the brain that are helpful for gaining explicit knowledge of implicitly acquired regularities.

## Experiment 1

**Figure 2.**
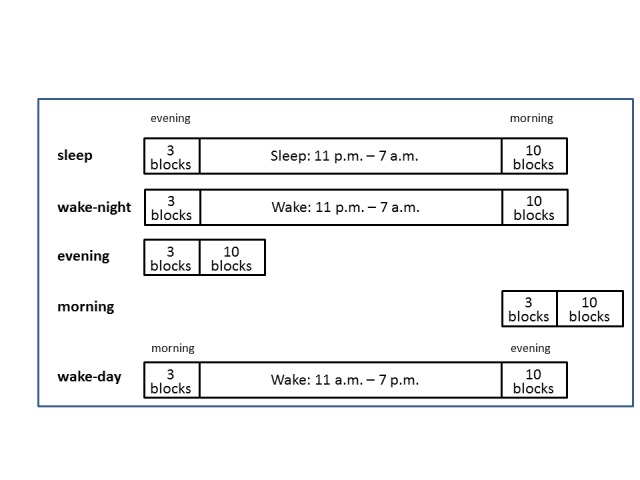
The five groups tested in Experiment 1 (Wagner, Gais, Haider, Verleger, & Born, 2004).

This experiment (Wagner et al., 2004) made a global test for benefits of sleep for attaining insight in the NRT. The task consisted of three phases: There was a first encounter with the task in three blocks, which might be followed by an incubation phase, followed by another session with the task in 10 blocks (or less, if insight was attained). Five groups were tested (Figure 2), differing by the presence and kind of incubation phase between the first and the second session: Three groups had an 8-hr incubation phase spent either asleep during night-time or awake during night-time or during day-time (*n* = 22 in each group). Two other groups lacked the incubation phase and served for controlling for beneficial effects of performing either in the evening or after sleep in the morning (*n* = 20 each). Participants were university students, aged 18-32 years, with equal numbers of men and women in each group.

### Task and procedure

The NRT task was used as described above (Figure 1). The “1,” “2,” and “3” keys on the PC numeric pad were labeled as “1,” “4,” and “9,” and served as response keys. There were 30 trials in each block. Thus, the first session consisted of 90 trials. Five of the 106 participants discovered the covert rule in these blocks already and were excluded from analysis and replaced by new participants. The second session consisted of 10 blocks, summing to 300 trials. However, when a participant attained insight, indicated by 10 successive correct shortcuts in one block, the task was terminated.

As control variables, participants indicated their levels of tiredness, activation, concentration, and motivation on 5-point scales immediately before either NRT session.

### Results and discussion

**Figure 3.**
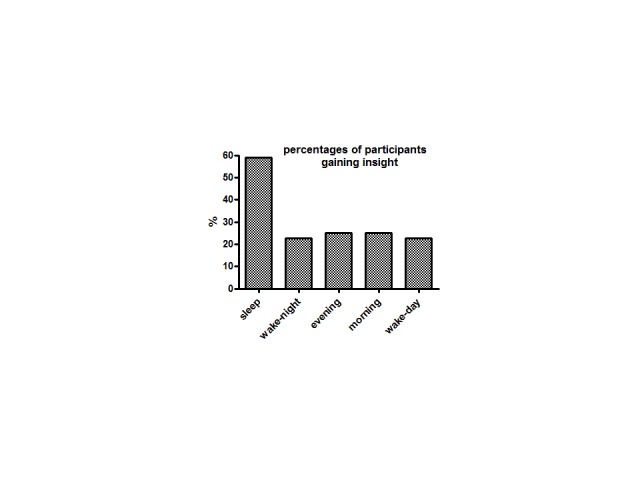
Percentages of participants gaining insight in the second session.

The major result is depicted in Figure 3: Of all participants in the sleep group, 59% (13/22) attained insight, which was reliably more than the five participants in each of the four other groups (out of 22 = 22.7% or out of 20 = 25%), yielding χ^2^ = 10.2 (*p* = .03) in a global test on all five groups (*df* = 4).

So it appears from this experiment that there is a constant probability of about 20-25% that some participant will attain insight in this task, and that there is an additional effect of sleep over and above this base rate, leading to insight in a further proportion of about 30-40% of participants. The next two experiments will shed some light on each of these two branches: on the one hand - on the determinants of the 20% base rate, and on the other hand - on the mechanisms of the sleep effect.

## Experiment 2

In this experiment (Lang et al., 2006), participants performed the NRT in one long session of 432 trials (eight blocks of 54 trials). We attempted to find precursors of insight by measuring event-related EEG potentials (ERPs) during the task. When planning the study, our main idea was to distinguish between stimulus-related and response-related ERP precursors. Therefore the task was modified, in order to unambiguously define the event to which a given ERP was related. In hindsight, this distinction was less relevant. What turned out to be relevant was the global ERP response to the display of the task.

### Task and procedure

Data from 26 participants could be used for analysis. For the reason stated above, unambiguously defining the event to which a given ERP was related, the NRT was modified. Trials started 500 ms after a warning signal, by presenting only the first pair of the stimulus string. After the participant had entered the response, which appeared below the stimulus string as the first member of the response string, the next digit of the stimulus string appeared 500 ms later. This way, not only the response string built up during the trial from left to right, but the stimulus string as well, rather than already being presented in its full length at trial onset. Because attaining insight into the digit structure might be more difficult when both strings are presented digit by digit, the length of the strings was reduced by 2, from eight in Experiment 1 to six for the stimulus string, and therefore from seven to five for the number of responses. The mirrored structure of the response string to be discovered had the general form A-B-C-C-B (with A, B, and C representing one of the digits 1, 4, or 9) - that is, the last two responses always mirrored the preceding two responses, so that again the second response in each trial was identical to the final result. EEG was recorded from 17 recording sites, and averaged across trials of the first block of the session and across trials of the block before insight was attained by rule-discoverers (with the actual block number varying across participants, of course) and for the same blocks in non-discoverers (with the actual block number individually yoked to rule-discoverers).

### Results and discussion

Of the 26 participants, six (= 23%) attained insight, as evidenced by their consistently pressing the “Enter” key already after the second response. This occurred between Blocks 4 and 8, with the median at Blocks 6 and 7.

**Figure 4.**
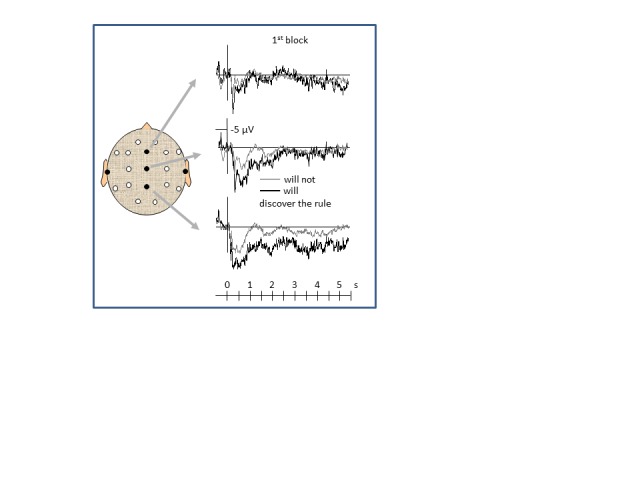
Grand mean ERPs of the first block during the first seconds of each trial, separately averaged across the six participants who will later discover the rule (black lines) and those 20 participants who will not (gray lines). Time point zero denotes presentation of the first digit pair, 500 ms after the warning signal. Negativity is plotted upwards.

Grand averaged ERPs evoked in the first block of the task are displayed in Figure 4.

In this first block already, ERPs differed between the group of six participants who would later discover the rule and the 20 participants who would not. The later rule-discoverers had larger parietal positivity, *F*(1, 24) = 5.6, *p* = .03, for the mean value of the first 5 s at Pz, or *F*(1, 24) = 5.1, *p* = .03, for the first second (0.2-1.2 s) at all three midline recordings displayed in Figure 4. This may be classified as a P3b evoked by the presentation of the first digit pair because this is an informative, task-relevant event (Sutton, Braren, Zubin, & John, 1965) which then evolves into a slow positive wave. We have suggested that P3b reflects the degree of internal monitoring of whether the stimulus is correctly processed (Verleger, 2008; Verleger, Jakowski, & Wascher, 2005), so its enhancement may mean here that the rule-discoverers pay more attention to their processing of the digit pairs. The ensuing slow positive wave might reflect the continuation of this monitoring process with the following stimuli and responses or, alternatively, as we argued in Lang et al. (2006), may reflect encoding to short-term memory, as was shown for slow positive waves at posterior sites by Ruchkin, Johnson, Canoune, and Ritter (1990), by Rösler and Heil (1991), and by others. Whatever the precise interpretation of these ERP differences may be, these results suggest that the 20% of participants who are rule-discoverers in this task already differ from the other participants in the very beginning of the task by the way how they approach the task.

It remains unclear whether these differences in approaching the task were mediated by the relevance assigned by individual participants to the passage in the instruction that the “Enter” key might be pressed whenever the final result had been entered. This instruction was dropped in a follow-up study by Rose, Haider, and Büchel (2010) in a simpler version of the task, characterized by shorter strings, requiring four responses only, by a more evident regularity, with the final response being predicted by the very first response already, and by more salient symbols, using color squares rather than digits. In that easier situation, about 50% of participants discovered the rule, and differences between rule-discoverers and non-discoverers did not exist from the very start but rather developed during task performance (cf. also Wessel, Haider, & Rose, 2012). Rose et al. (2010) found that awareness for the regularity was preceded in rule-discoverers by an increase in neural activity (measured by functional magnetic resonance imaging [fMRI]) in the ventral striatum and the right ventrolateral prefrontal cortex, as well as by increased high-frequency coupling between distant brain areas (measured by gamma coherence in the EEG).

**Figure 5.**
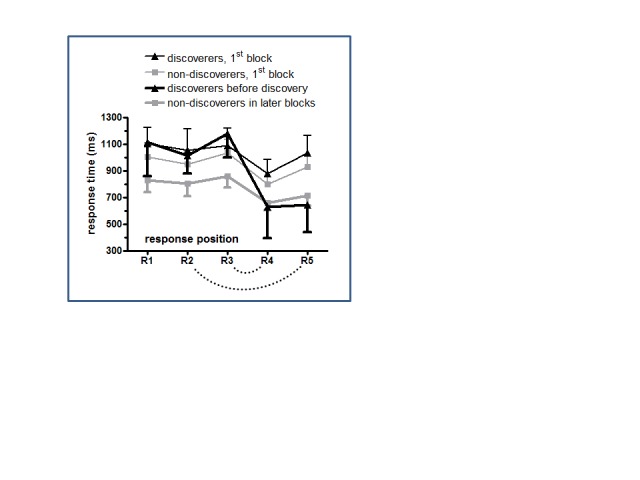
Means and 95% confidence intervals of response times across participants. Black lines denote rule-discoverers, gray lines denote non-discoverers. Thin lines denote the first block, bold lines denote the block before insight. The dotted arcs connect responses that are identical according to the covert rule (R3 & R4, R2 & R5).

Returning to our task, it is of interest that these group differences were visible in ERPs evoked in the first block even though response times for entering the responses with each pair did not differ between groups in the first block, F 1.3, *ns*, for effects of Group and Response Position × Group in ANOVA on the first block (thin lines in Figure 5).

Figure 5 shows that the non-discoverers did learn something else: All their responses became faster in the course of the task, in contrast to the discoverers who became faster specifically with the responses R4 and R5 that were predictable by the covert rule. The ANOVA on the response times depicted in Figure 5 yielded an interaction of Response Position × Block × Group, *F*(4, 96) = 6.8, GG-corrected *p* = .002, because although, as mentioned, there were no differences between groups in the first block, responses at the non-predictable R1, R2, R3 were faster in the later block in the non-discoverers than in rule-discoverers - Block × Group in separate ANOVA of the block before insight: *F*(4, 96) = 7.2, GG-corrected *p* = .002. This non-specific speeding may be called unspecific “procedural” learning and presumably refers to automation of applying the two arithmetic rules and of finding the response keys. Over and above this procedural learning, there was also some specific learning of the covert rule even in non-discoverers. In the later block, the predictable responses R4 and R5 were faster than the preceding unpredictable responses in both groups. While this may be trivial for R4 that always directly repeated R3, this was also true for R5 compared to the identical R2 in rule-discoverers in the block before insight, *F*(1, 5) = 10.2, *p* = .02, and tended to be true even in non-discoverers, *F*(1, 19) = 4.0, *p* = .06. Thus, in addition to unspecific procedural learning, there was also implicit learning of the hidden regularity to some extent in these non-discoverers.

## Experiment 3

As noted in discussing the results of the preceding experiment, implicit learning occurred to some extent even in participants who did not attain explicit insight into the hidden rule. In Experiment 3, non-conscious implicit learning turned out to play a relevant role for the beneficial effects of sleep on attaining insight in the NRT.

One major reason to conduct this experiment was to split the beneficial effect of whole-night sleep (cf. Experiment 1) into effects of deep SWS and REM sleep. Following Plihal and Born’s (1999) approach, the distinction between SW-rich and REM-rich sleep may be realized by using either the first or the second half of the night because there is more SWS during the first half and more REM sleep during the second half of night-sleep. Therefore, the two NRT sessions and the intervening sleep-filled incubation period took place either in the first or in the second half of the night. Based on a number of preceding studies (Diekelmann & Born, 2010), it was expected that hippocampus-dependent memories would be consolidated and reprocessed by SWS and that hippocampus-independent perceptual and procedural memories would be consolidated and reprocessed by REM sleep. At the time of planning the study, there was already evidence from fMRI studies on the NRT that the implicit acquisition of the regularity, indicated by response time differences between predictable and unpredictable responses, activates the perirhinal cortex and possibly the hippocampus, whereas procedural learning of the task, reflected in unspecific speeding of all responses, activates the basal ganglia (Rose et al., 2002; Rose, Haider, Weiller, & Büchel, 2004). Since insight is expected to benefit from implicit acquisition of the regularity, since this acquisition probably is hippocampus-dependent, and since hippocampus-dependent memories are probably reprocessed during SWS, it was expected that SWS would play a decisive role in sleep’s beneficial role for attaining insight, more so than REM-sleep.

Note that this assumed relevance of the hippocampus for implicitly acquiring and reprocessing the regularity of the NRT response patterns differs from the traditional conception which ascribed a role for “declarative” (i.e., consciously accessible and verbalizable) memories to the hippocampus. This traditional conception has been more and more controversially discussed in recent years (see e.g., Henke, 2010).

### Task and procedure

**Figure 6.**
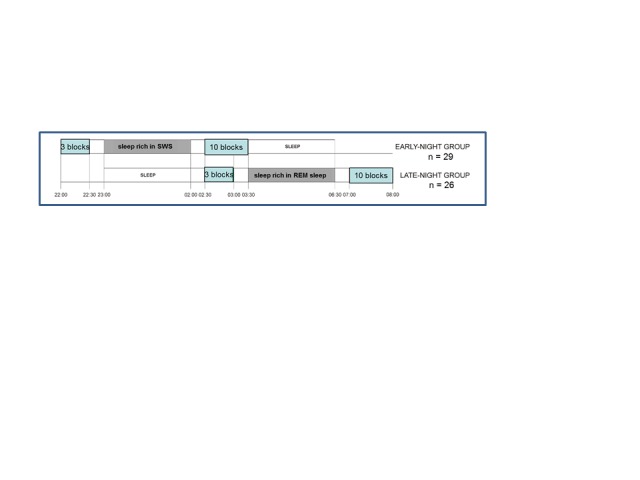
Design of Experiment 3.

The same version of the NRT was used as in Experiment 1, described above (Wagner et al., 2004). The design of the study is outlined in Figure 6. All participants had an adaptation night - that is, they got acquainted to the lab and the bed on a night before the actual experiment. Data from 55 participants could be analyzed.

Early-night participants reported to the laboratory at about 9:00 p.m., performed the first session of three blocks (including preceding computer-guided practice) at about 10:00 p.m. (after placement of electrodes for EEG and polysomnographic recordings), and thereafter went to bed at about 11:00 p.m. After 3 hr of sleep they were awakened to perform the 10 blocks of the retest. Late-night participants reported to the laboratory at about 10:00 p.m. and, after placement of electrodes, first slept for 3 hr (to “consume” SWS) before performing the first session at about 2:30 a.m. Then, they slept again for another 3 hr (about 3:30-6:30 p.m.), followed by retesting in the morning. Participants were awakened from shallow sleep Stages 1 or 2 only, to avoid cognitive disturbances that may occur when being raised out of SWS or REM sleep.

### Results for explicit insight irrespective of previous state

**Figure 7.**
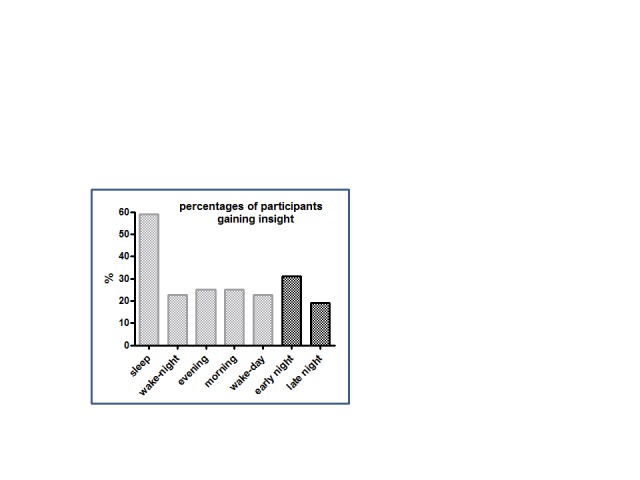
Percentages of participants gaining insight in the second session. The leftmost five bars are from Experiment 1 (see Figure 3) and the two rightmost bars are from the present Experiment 3.

Nine of the 29 early-night participants (31%) and five of the 26 late-night participants (19%) attained explicit knowledge of the covert rule after sleep (Figure 7).

This percentage of 19% in the late-night group is certainly not more than the percentage of 20% reached in the wake-control groups of Experiment 1. The percentage of 31% in the early-night group may tend to exceed those wake-control groups (though did not significantly so) and is certainly much less than the percentage of explicit rule-discoverers in the whole-night sleep group of Experiment 1.

We attribute this lowered percentage of rule-discoverers compared to whole-night sleep to the stressful circumstances of being roused in the middle of the night and possible complementary beneficial effects of whole-night sleep, with its sequence of SWS and REM phases (see Gais, Plihal, Wagner, & Born, 2000, for similar beneficial effects of whole-night sleep vs. half-night sleep). As a consequence of this low percentage, the beneficial effects of sleep that we did find in the following may be an underestimation of the effects that would normally prevail when sleep extends uninterruptedly through the whole night.

### Transitions from implicit to explicit knowledge

**Figure 8.**
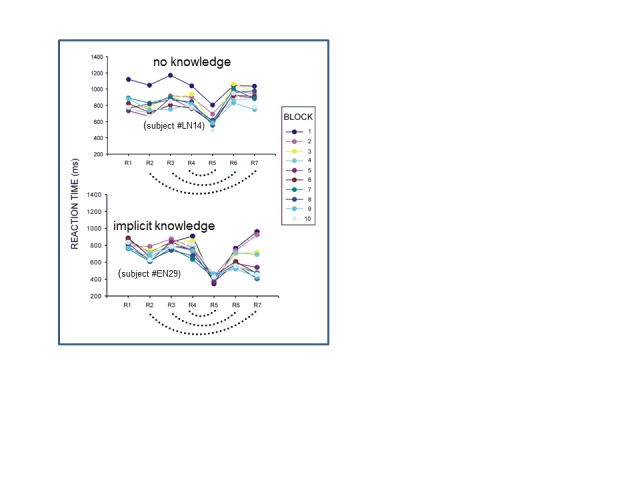
Illustration of how participants were classified as having or not having acquired implicit knowledge of the covert rule. Depicted are mean response times over the 30 trials of each block, separately for each of the 10 blocks in the second session for the seven responses R1 to R7 within a trial. Participant EN29 (lower panel) had faster response times for R6 and R7 than for R3 and R4 from Block 3 onwards and therefore was classified as implicit learner, in contrast to Participant LNLN14 whose data are depicted in the upper panel. The dotted arcs connect responses that are identical according to the covert rule (R4 & R5, R3 & R6, R2 & R7).

Each participants’ response times were individually tested for each block whether they did or did not show a pattern of implicit learning, defined as mean response times of the predictable responses R6 and R7 being faster than mean response times of the unpredictable responses R3 and R4 (cf. Figure 8), accepting an effect only if *p* < .01 (with the 30 trials of each block providing the error variance). This classification was done independently for the blocks before sleep and after sleep. In detail, if at least the second or third of the three blocks before sleep was classified as displaying implicit learning, then the participant was classified as “implicit learner before sleep” (impl-pre). Similarly, if at least three consecutive blocks of the 10 blocks after sleep were classified as displaying implicit learning, the participant was classified as “implicit learner after sleep” (impl-post).

**Figure 9.**
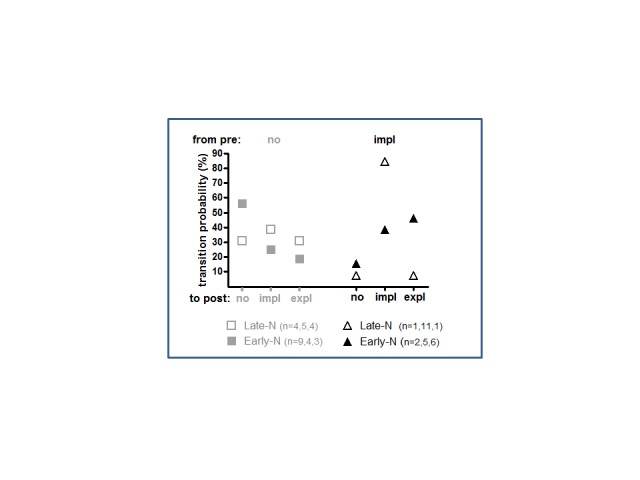
Frequencies of transition from knowledge stages in the first session to knowledge stages in the second session. Knowledge stages in the first session are: no knowledge (grey, left side) or implicit knowledge (black, right side). Knowledge stages in the second session are: no, implicit, or explicit knowledge (from left to right on the *x*-axis). Solid symbols are for the early-night group, dashed symbols for the late-night group. The indicated percentages depict the number of participants in each of the three sub-groups (no-, impl-, expl-post) relative to their total number.

We subdivided all 55 participants according to whether their response times in their first session (“pre”) did or did not reflect implicit knowledge of the covert rule (“impl-pre” or “no-pre”). The late-night group split to *n* = 13 impl-pre and *n* = 13 no-pre participants, and approximately the same was true in the early-night group (*n* = 13 impl-pre and *n* = 16 no-pre). Next we examined what became of each of these sub-groups in their second session (“post”): Whether they had attained insight (“expl-post”) or showed implicit knowledge (“impl-post”) or no knowledge (“no-post”). Results of this analysis are displayed in Figure 9.

Participants with no knowledge before sleep (grey symbols in Figure 9) did not display preferences in their transition to learning-stages after sleep - that is, it was equally likely after sleep that they would not display any signs of knowledge or acquired implicit knowledge or even attained insight (χ^2^ = 1.9, *p* = .38, with *df* = 2) and this was also true when these three post-subgroups were subdivided in early- and late-night subgroups (χ^2^ = 1.9, *p* = .39, with *df* = 2; cf. Figure 9). However, things were different in participants who had displayed implicit know-ledge already before sleep. These participants were unevenly distributed also after sleep because, perhaps not surprisingly, only very few of them fell back to no knowledge: three no-post, 16 impl-post, seven expl-post (χ^2^ = 10.2, *p* = .006, with *df* = 2). What was not trivial is that this distribution additionally differed when these three subgroups were subdivided in early- and late-night subgroups (χ^2^ = 6.2, *p* = .046, with *df* = 2). Whereas in the late-night group, the bulk of participants stayed with their implicit knowledge after sleep, in the early-night group, an appreciable amount of participants made the transition from pre-sleep implicit knowledge to post-sleep explicit knowledge.

### The role of sleep for the transition from implicit to explicit knowledge

**Figure 10.**
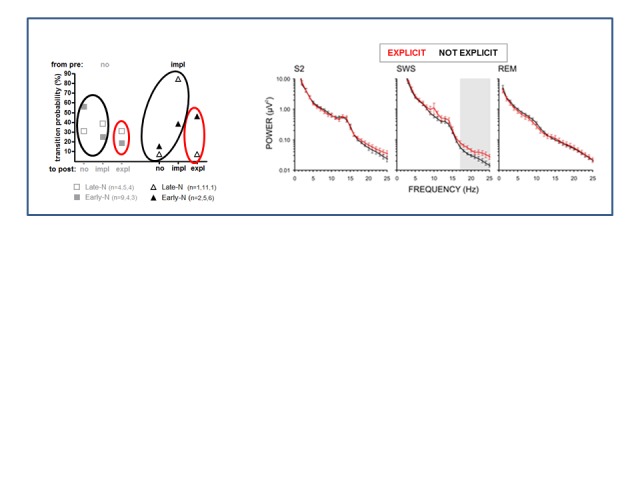
Power spectrum of sleep EEG for explicit rule-discoverers versus all other participants. The left part of the figure repeats Figure 9, in order to clarify which subgroups’ data are depicted, by encircling the participants who contributed to black and red values in the power spectrum (right part) by black and red ellipses, respectively. Discoverers are 13 “expl-post” participants (in red), non-discoverers are all other 33 participants (in black). (EEG could not be analyzed in one of the 14 expl-post participants and in eight of the other 41 participants.) Depicted is the grand average power spectrum (across electrodes C3 and C4) for three sleep stages: S2, SWS, and REM. Shaded area in SWS indicates the frequency range of significant differences between non-solvers and solvers. Standard error bars are presented for each frequency bin.

**Figure 11.**
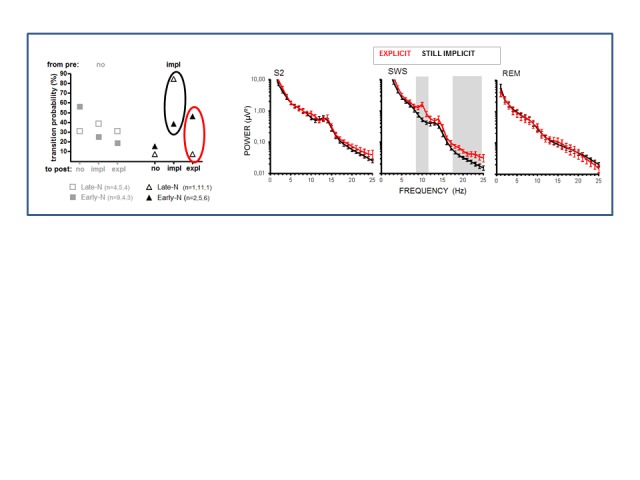
Power spectrum of sleep EEG in participants who displayed implicit learning before sleep. Explicit rule-discoverers (red, *n* = 7) are compared to participants who maintained implicit knowledge in the second session (black, *n* = 14). See Figure 10 for further details.

**Figure 12.**
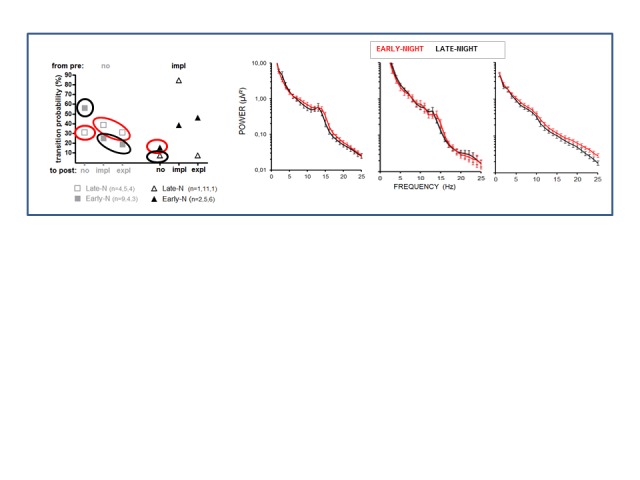
Control analysis for effects of early- (red, *n* = 15) versus late night (black, *n* = 10) on the effects depicted in Figure 11. See Figure 10 for further details.

We had a small window open for looking at processes occurring during sleep, having placed electrodes at left and right central scalp (C3 and C4), originally for making the traditional classification of sleep-stages according to Rechtschaffen and Kales (1968). This EEG recorded during sleep was transformed to the frequency domain by the Fast Fourier Transform of epochs of 5.12 s, and these frequency spectra were averaged across epochs separately for sleep stages S2, SWS (S3 and S4), and REM. Power was measured in windows of 1 Hz. These power values were compared within frequency bands between the sleep-transition subgroups. Results are displayed in Figures 10-12.

Figure 10 displays the comparison between explicit rule-discoverers and all other participants. Insight after sleep was associated with higher beta power in SWS, *F*(1, 42) = 6.8, *p* = .01, in ANOVA on the averaged beta band (17-25 Hz). This effect did not differ between early- and late-night groups.

Figure 11 restricts the comparison made in Figure 10 to those participants (impl-pre) whose behavior reflected implicit knowledge before sleep and who either stayed on this level after sleep or dis-covered the rule. The effect on beta power in SWS found in the preceding comparison also held in comparison of these two subgroups, *F*(1, 19) = 6.1, *p* = .02. Additionally, a specific effect emerged in this comparison only, as larger power in the alpha band (average power of 8-12 Hz) for those impl-pre participants who would discover the rule after sleep, *F*(1, 19) = 4.4, *p* = .05, in particular in the 10 Hz and 11 Hz frequency bins (*p* .04 in analyses on single frequencies).

It may be suspected that this new effect in Figure 11 is confounded by a difference between early- and late-night participants because more rule-discoverers are from the early-night group and more participants who stay implicit are from the late-night group. Therefore, Figure 12 depicts, as a control analysis, the comparison between early- and late-night participants in all participants not included in Figure 11. No effect of early- versus late-night was significant.

## Discussion

By applying the NRT at two consecutive sessions separated by an interval, we investigated the role of sleep in this interval for attaining insight at the second session. A night of sleep was shown to triple the number of participants attaining insight above the base rate of about 20%. The 20% of participants who formed the rate of rule-discoverers also in a second study differed from other participants in their task-related EEG potentials from the very beginning already, independently of sleep. In the third study, one mechanism effective during sleep in raising the number of insightful participants above this base rate was an effect of the deep-sleep phase of SWS on participants who had implicitly acquired the covert regularity before sleep. In the following, we will highlight the significance of these sleep-EEG results and refer our findings to other studies about the relation between sleep and creativity.

### Sleep-EEG results

EEG characteristics of SWS had predictive value for explicit know-ledge generation after sleep: Beta power (17-25 Hz) during SWS was increased in participants who would discover the hidden structure of the NRT after sleep (expl-post). Moreover, among these expl-post rule-discoverers, power in the alpha band (10 Hz) was increased in SWS in the impl-pre participants - that is, in those participants whose response times of the session before sleep indicated implicit learning of the fact that the sixth and seventh responses were predictable.

In waking participants, beta power increase has been assumed to reflect enhanced local synchronization of functionally specified locally distributed networks (Pfurtscheller & Lopes da Silva, 1999). The current increase of future rule-discoverers’ beta-power in SWS may therefore indicate stronger synchrony of local networks during their SWS. How this may lead to insight in the session after sleep remains unclear. Synchronization of a widely distributed long-distance beta network was reported to be a marker of conscious access to the content of verbal material when neuronal responses to briefly flashed words were compared between masked and unmasked words (Gaillard et al., 2009). In this line of thinking, it may be speculated that this enhanced beta during SWS was a precursor of some enhancement of beta power during task performance that might be relevant to conscious insight, but additional analyses provided no evidence for differences between rule-discoverers and non-discoverers in beta power during task performance, both before and after sleep (Yordanova et al., 2012). We may note that a decrease (rather than an increase) in beta power was related to insight in solving brain-teasers (Sheth et al., 2009), but the situations may not be comparable between that study and the present one.

Most intriguing was the increase of 10-12 Hz power of the EEG recorded during SWS in participants who converted pre-sleep implicit knowledge to post-sleep explicit insight. This frequency range of 10-12 Hz is lower than the frequency ranges reported traditionally for slow (~12 Hz) and fast (~14 Hz) sleep spindles (e.g., De Gennaro & Ferrara, 2003; Gibbs & Gibbs, 1950). However, more recently, the 10-Hz component, with a fronto-central topography, has been described as an essential component of SWS (Duckrow & Zaveri, 2005; Salih et al., 2009). Some recent studies have provided links between such 10-12 Hz activity during SWS and memory. Marshall, Helgadóttir, Mölle, and Born (2006) stimulated sleeping participants’ scalp by slowly oscillating direct current (0.75 Hz) and found that 8-12 Hz EEG power during SWS (with a maximum at 10 Hz) was specifically enhanced by this stimulation and was correlated to improvements in recalling pairwise-associated words after sleep. Griessenberger et al. (2012) found that an increase of the 8-12 Hz band during sleep spindles in SWS was correlated to better retention of the order of objects that had been memorized before sleep when memories were tested three days later (cf. also Cox, Hofman, & Talamini, 2012, although their focus was on the 11-16 Hz band).

It might be suspected that these increases of beta power and of 10-12 Hz power in future rule-discoverers reflect epochs of heightened arousal, thus of lightening of SWS, and that such nearly-awake epochs might be helpful for attaining insight after sleep. In particular, the 10-12 Hz activity might reflect alpha activity. Alpha is defined as EEG activity in the 813 Hz band during a relaxed waking state, with its topographical focus at occipital scalp sites. Undoubtedly, the 10-12 Hzfrequency falls within the alpha band. However, it is improbable that participants were awake, because the effects were specific to SWS and were absent during S2 and REM. In contrast, arousal from sleep, as possibly indicated by alpha, should have more distinct effects in lighter sleep stages: It should be more probable in S2 than in SWS that lightening of sleep should lead to nearly awake phases reflected by alpha intrusions. Moreover, EMG activity during SWS was not higher in rule-discoverers than in non-discoverers, although an increase of EMG activity would be expected in case of more microarousals. The other criterion for distinguishing alpha from the above described 10 Hz activity during sleep is topography. Alpha activity is always largest at posterior scalp sites whereas the 10-12 Hz activity occurring during sleep has a frontal focus. Since we had data from only two recording sites in the present study, the topographical focus was impossible to determine. However, we were able to replicate the rule discoverers’ larger 1012 Hz activity in a new data set (still unpublished; this time using the SRTT) where sleep EEG was recorded with a full montage and the 10-12 Hz activity had a distinct fronto-central focus. Thus, it seems plausible to relate the 10-12 Hz effect during SWS to slow spindle activity, which leads to the following interpretation of this finding.

The compound of hippocampal ripples and cortical spindles as observed in sleeping and resting rats probably reflects information transfer from the hippocampus to the neocortex (Siapas & Wilson, 1998; Wierzynski, Lubenov, Gu, & Siapas, 2009). Similar observations have been made in humans, with hippocampal ripples being measurable in intracranial recordings from patients (Axmacher, Elger, & Fell, 2008; Mölle, Eschenko, Gais, Sara, & Born, 2009). But even in intact humans, activation in the hippocampus could be measured during SWS by means of fMRI, in response to specific task-related cues presented to the sleeping participants (Rasch, Büchel, Gais, & Born, 2007; van Dongen et al., 2012). It has also been shown by fMRI measurements that the medial temporal lobe including the hippocampus is involved in implicit learning in the NRT. As mentioned above, this had already been shown some time ago by Rose et al. (2002, 2004) for the medial temporal lobe. More recently, this finding has been confirmed for the hippocampus proper in the NRT (Darsaud et al., 2011) and in the SRTT (Rose, Haider, Salari, & Büchel, 2011).

Thus, it may be speculated that the oscillatory 10 Hz patterns in the implicit-to-explicit converters’ sleep-EEG during SWS reflect read-out of hippocampally stored information about the implicitly learned relationships to the neocortex. Such read-out implies some restructuring of this information, which might increase the probability of interactions with explicit processing systems, thereby possibly leading to explicit knowledge about the implicitly acquired relationships.

### Sleep and creativity

As noted in the Introduction section, insight into a hitherto unidentified regularity may be considered a particular type of creative behavior. The beneficial role of sleep for creative processes has been experimentally confirmed by at least three independent studies so far: by our Experiment 1 (Wagner et al., 2004), by Cai et al. (2009), and by Ritter, Strick, Bos, van Baaren, and Dijksterhuis (2012). Ritter et al.’s study is of general interest by its use of task-related odors during sleep for boosting creative solutions to this task in the next morning (cf. Rasch et al., 2007). Of relevance in the present context, Cai et al. reported that REM sleep during an afternoon nap led to increased use of words encountered in another context before sleep as solutions in the remote association test after sleep. The authors concluded that “REM sleep is important for assimilating new information into past experience to create a richer network of associations for future use” (p. 10333). These results might be interpreted to be in conflict with the emphasis on SWS in the results of our Experiment 3 (Yordanova et al., 2008, 2012). But actually there is ample room for both results within a more general framework. Cai et al.’s results, favoring REM sleep stage as important for attaining insight, rightly caution against the nearby interpretation of our results that SWS is more relevant to attaining insight than REM sleep. Indeed, this conclusion cannot be drawn from our data: There were not significantly more participants in the (SWS-rich) early-night group than in the (REM-sleep rich) late-night group who gained insight (see Figure 7), and in any case the percentage of rule-discoverers in the (SWS-rich) early-night group was appreciably lower than this percentage in the (SWS- and REM-rich) whole-night group (Figure 7). It is true that our data enabled us to describe a way to insight that leads via SWS, from implicit learning before sleep via the enhanced processing in the 10 Hz range during SWS. But, obviously, there were still other ways to attain insight, as is evident from the equally large number of participants who attained insight after sleep without having implicitly acquired the predictability of the final responses in the session before sleep (Figure 9, left panel).

Possibly, the choice of tasks has already predetermined the differences in processing preferences for sleep stages between Cai et al.’s (2009) and our studies. In research on creativity, tasks may be classified as requiring either divergent thinking or artistic processes or insight, with insight tasks being more narrowly defined than those of the other two domains with respect to the creative solution appropriate to the task (Dietrich & Kanso, 2010, p. 823). Using this classification, Cai et al.’s task is typical of divergent thinking and our insight task is characteristic of a more narrowly defined task. Modes of processing might a priori differ between these classes of tasks. Based on this distinction, it might be speculated that the SWS path to insight that we have described is the more logical, analytical, “left-hemisphere” mode, whereas the REM path to insight (suggested by Cai et al.) is the more intuitive, integrative, “right-hemisphere” mode. In any case, all these studies agree that sleep may foster creative thinking.

We did not make any attempts to link these assumed processes of restructuring during sleep to the subjective experience of dreaming during sleep (but see Wamsley, Tucker, Payne, Benavides, & Stickgold, 2010). Studies on subjective reports of mental contents when awakened from non-REM and REM sleep early or late in the night strongly suggest that these contents are more directed and focused in non-REM sleep in the first night-half, and more hallucinatory in REM sleep throughout the night (Fosse, Stickgold, & Hobson, 2004). This distinction nicely parallels the distinction just drawn between creative thoughts fostered either by SWS or by REM sleep. As is commonly known, mental contents during sleep are often not remembered any more in the morning. But the present research, in line with other research on sleep and memory (Diekelmann & Born, 2010; Stickgold & Walker, 2013), suggests that these contents, or at least their neurophysio-logical underpinnings, do affect mentation and behavior on the very next day and thereafter.
